# Round the Hospitals

**Published:** 1918-06-08

**Authors:** 


### ROUND THE HOSPITALS.

The intensity of the struggle now in progress in France, the danger to Paris, and the sense of overpowering force relentlessly bent on destruction, affect the entire civilised world. In all directions nurses are finding recovery retarded by the tension. Natures enfeebled by long nerve-strain feel it most. But there are many week-kneed folk who have not this excuse, who allow their spirits to fluctuate with the bulletin of the hour from the battlefield, and are ever ready to succumb to the worst forebodings and force them on whoever will listen. There can be no question that those bear the strain best who hold the safety of others in their hands. Spiritual force alone can fortify the mind against the contagion of apprehension set floating in the air at times when the public nerve

**ROUND THE HOSPITALS**—*(continued)*.

is tested as it is this week. And those whose lives are being spent for others keep open the door of their minds in that very act to the influx of spiritual strength. The forces which are set in operation by mechanical means, the long-range gun, the bomb, the terrific pressure of numbers, are poor indeed when confronted with a mind power reflected from the Creator. And there is nothing so formidable in the whole army of the Kaiser as the immovable resolution of the men and women opposed to it. To maintain our own confidence unshaken, to sustain our brothers and sisters with a strong faith in their ultimate triumph, undismayed by evil tidings, serene even in disaster, is the appointed way to help. A chain of devoted minds linked each to each with unswerving resolution, this is the really formidable weapon. Through this we shall conquer all foes and win through to Victory.

EVEN as we go to press the College of Nursing is holding its memorable annual meeting, the first in which members of the Council will be declared returned by open nomination and election. It is an event of the first importance in the nursing world. A rising tide of enthusiasm has caught the College, stimulated rather than hindered, so it really appears to us, by a measure of transparently insincere opposition, and nurses have now a clear issue before them. The unexpected has happened. The cause of nursing reform has fallen into the hands of a wise and disinterested statesman, who not only knows what things need to be done, but possesses power to push them through. By means of the confidence Sir Arthur Stanley, with the aid of Sir Cooper Perry and other able supporters, has been able to inspire, he has brought into common agreement the three forces it was necessary to enlist for the reorganisation of nursing affairs—viz., the governing authorities of the voluntary and municipal hospitals, the matrons responsible for the training of nurses, and the nurses themselves. With rare insight he has judged that the matters affecting strictly professional interests can only properly be decided by members of the profession themselves. These leaders have done, and are doing, for the nursing profession that which it was unable, without their aid, to accomplish for itself. The College of Nursing has rescued the situation and set nurses free to rule their own house. So much has been done already, but this annual meeting is an earnest of much more in the future. In times to come nurses will reckon it one of their claims to distinction that they were members of the College and many of them present on this historic occasion. For this meeting, held in one of the darkest weeks of the war, marks the fact that union among British nurses is now becoming a reality and means triumph for the profession of nursing.

MATRONS and Sisters are included in His Majesty's Birthday Honours List which was published on June 3. The Royal Red Cross has been conferred on the following ladies:—*First Class*: Miss Margaret E. Goodall-Copestake, superintending sister Q.A.R.N.N.S., and Miss Muriel G. Hutton, nursing sister Q.A.R.N.N.S. Reserve. *Second Class*: Miss Sarah E. McClelland, nursing sister Q.A.R.N.N.S., Miss Vera L. Spark and Miss Zoe E. Stronge, nursing sisters Q.A.R.N.N.S. Reserve; Miss M. C. Tod, matron and masseuse, R.N. Auxiliary Hospital, Corstorphine, Edinburgh; Miss E. C. Philp, matron, Northern Infirmary, Inverness; Miss E. K. Finnemore, matron, Queen Mary's R.N. Hospital, Southend; Mrs. E. Ritchie, matron, Auxiliary Naval Hospital, Grangetown, Yorkshire; Mrs. A. M. England, staff nurse, R.N. Hospital, Mount Stuart, Isle of Bute; Miss Isabella Frame Lang, theatre sister, R.N. Hospital, Mount Stuart, Isle of Bute. The "Kaisar-i-Hind Medal for Public Service in India" of the First Class has been bestowed on Sister Mary Victoria, Clewer Sisters of St. John the Baptist, Principal, Diocesan College for Indian Girls at Ballygunge, Calcutta.

WE learn that no fewer than fifty-five nurses of the Canadian Military Nursing Service won the Mons Star, nurses to the number of 104 having come over from Canada in September 1914, some to England, some to France, and having begun nursing in France by November in that year. One of the features of the Canadian Service is that it contains no staff nurses. All are either sisters or matrons. As such they hold the rank and pay of officers in the Canadian Military Service. The Matron-in-Chief, Miss Macdonald, holds the rank of major; other matrons rank as captain, and the sisters as lieutenant. The brass buttons worn by these nursing officers have the maple wreath instead of the laurel worn by the army officers of the Dominion. The Canadian sisters have done admirable work in many parts of the world since the early days of the war, and now number some 1,900, who are distributed mostly in France and England. Dozens of hospitals in England have been equipped by Canada, and are wholly staffed by the Canadian Military Nursing Service, some of the best known and largest establishments being at Epsom, Orpington, Basingstoke, Taplow, Buxton, and Brighton. There is a beautiful little convalescent hospital for the Canadian sisters at Buxton, containing forty beds, and further accommodation for them at Bushey Park and Wokingham.

WE regret to record the death on May 20, after a brief illness, of Sister Edith Hughes, for sixteen years in the employ of the Chester Board of Guardians, during ten of which she held the office of superintendent. Since the military authorities took over the hospital, Sister Hughes has been sister in charge of E Division, and she may be said to have died at her post. She was very warmly esteemed by her colleagues and by the Guardians, and most popular with her patients. She was

**ROUND THE HOSPITALS**—(*continued*).

buried with military honours, and her funeral attested the appreciation of the town for her long-continued labours among the poor and recently among the wounded. The coffin, covered by the Union Jack, was borne by six convalescent patients from the hospital. Colonel Prout, C.M.G., R.A.M.C., Officer Commanding the Military Hospital, and Miss Spencer-Jones, A.R.R.C., matron, followed after the chief mourners, with many of the medical and nursing staff from the War Hospital, the Chairman of the Board of Guardians, and a large company of those who had formerly worked with the deceased sister. The first part of the burial service was read in the hospital chapel, whence a long procession was formed to Guilden-Sutton Church, headed by the Castle Depôt Band, and accompanied by a detachment of the N.C.O.s and men of the R.A.M.C. Soldier patients formed the firing party and bugles sounded the " Last Post."

THERE can be little doubt that the remedy for V.A.D. shortage in auxiliary hospitals situated near towns or large villages lies in enlisting those who can give part-time only. This system, wherever tried with practical ability on the part of the matron, has proved eminently satisfactory, and we find the resident medical officer of the Livingstone College Hospital in the East End strongly recommending it. For one woman who can give whole-time, or even part-time, every day to hospital work, there are at least a hundred who could give four or five hours some two or three times a week. All that is needed to ensure efficiency is a schedule of workers with their hours and days, a few names of volunteers willing to take emergency work—for these part-time workers are subject to unexpected family claims—and a certain degree of patience in initiating new workers into their routine. We have watched this system work with the greatest smoothness year after year, since war began, in a small town near the coast, the greatest anxiety being experienced among the workers not to " lose their job " through pressure of other volunteers eager to take a share. In fact, we have been led to place great faith in the part-timer, and to wonder whether the system might not be still farther extended to domestic work in civilian hospitals.

THE Purey-Cust Nursing Home in York is one of the oldest organisations for providing trained nurses. It began in 1870 as an association for district nurses, and now comprises both a private nursing staff and a nursing home for persons of moderate means. The private nurses work on a " modernised system," presumably receiving their earnings less a percentage for expenses, and are said to be well satisfied with the arrangement. It is always difficult in enterprises of this kind to adjust the balance sheet. Part of the work is commercial, on the basis of a reasonable remuneration for services rendered. Part of it is purely philanthropic. The managers feel they must not make profits, and any surplus they receive is spent on the district nursing. The result of this is that small deficits are very likely to make their appearance, and this year the deficit on the Home amounts to £100, which it is hoped to obviate for the future by a slight increase in the charges made. The Dean of York, who took the chair at the annual meeting, was a little hard on the promoters of the annual sale, which was started as long ago as 1878, and this year realised the record sum of £150. He objected to such sales, which, he said, were very apt " to degenerate into bazaars," and thought the charity should be supported by direct subscriptions. As an abstract proposition few would gainsay this opinion. But a sale which dates back for forty years has a dignity of its own, and it enlists a whole army of willing workers unable to make gifts of money.

FROM Melbourne comes news that the Royal Victorian Trained Nurses' Association has had under consideration a motion for equalising nurses' fees so as to fall into line with Queensland, Western Australia, and New South Wales. It was settled that the uniform charge for all private nursing should be £3 3s. per week, with an extra charge of 5s. per week for infectious cases involving disinfection of clothes. Hitherto the charges have been £3 8s. 6d. for mental and alcoholic, fever and infectious cases, including phthisis, and for the first week of major operations. For midwifery the standard charge was £2 18s. 6d. The agitation for better pay and conditions for nurses in training continues, and an echo of it has reached Parliament, where it was asserted that nurses object very strongly to the fourth year in hospital imposed by the regulations. The National Council of Women has decided to petition the Government to appoint a Royal Commission to inquire into the whole question of the training of nurses. Meantime the State Executive has passed new regulations respecting the registration of midwives. For the future twenty-three is fixed as the minimum age at which registration under the Midwives Board can be granted.

WE have before alluded to the distressing illness and death of a probationer at the Melbourne Hospital just as she was nearing the completion of her third year. The additional facts which have come to light entirely exonerate the hospital authorities from blame in connection with the poor lady's mental and physical breakdown. But the incident has been laid hold of to make an attack upon the system of training at the Melbourne Hospital, and particularly on the comparatively new regulation increasing the training to four years. The hospital committee report that they had adopted the longer period of training partly because they found that three years " was not sufficient to ensure complete efficiency," and partly because, without the fourth-year or " staff " nurses, the proportion of untrained material in the hospital was too large. They report also that the conditions under which nurses are trained are infinitely

**ROUND THE HOSPITALS—(*continued*).**

superior to those existing before the new institution was built, that the nursing staff has been increased during the last seven years by forty-five—though the patients have only increased by forty—and that the domestic staff has almost doubled, thus relieving the nurses of much purely manual labour. They assert, moreover, that the number of hours nurses are on duty in the wards is "considerably less than those of English hospitals." For after administrative work the fourth year, when divorced from an inadequate salary and all trace of sweating, providing it is accompanied by advanced training for the higher posts, may prove of great value to educated and capable women.

Many of those present at the Nurses' Memorial Service at St. Paul's have wished for some memento of the occasion. We learn that the Stereoscopic Company, 3 Hanover Square, W., have published a beautiful souvenir of the service in the form of a photograph of the interior of St. Paul's Cathedral, under which, in the handwriting and with the signature of Canon Holmes, Archdeacon of London, are the following words: "In remembrance of the Memorial Service for Nurses who died for their country, St. Paul's Cathedral, April 10, 1918. Not one of them is forgotten before God." One hundred copies have been presented by the Rev. A. Lombardini to certain representatives of the nursing profession present on the occasion, and further copies can be obtained from the Stereoscopic Company at cost price, 2s.

We have been much struck by the interesting letter from "Unseen War Worker" which we publish this week in the Editor's Letter Box (see p. 214). It reveals very clearly where the shoe pinches in hospital domestic work. It does not "count," patriotically speaking, even in military establishments. As at present organised it is uninspiring, drudging, poorly esteemed. We depend for all the smooth working of the machine on these domestic helps, but we do not set out to make them understand how much we appreciate them. We want women who are alert, capable, devoted to duty, willing to spend themselves in our service. And we do not realise that the way we deal with this particular set of workers does not attract the alert type of women, it discourages them; it only draws in the poorer-spirited. "Unseen War Worker" makes a very sensible suggestion when she appeals for a uniform, such as that worn by V.A.D. General Service Members, with a medal for younger workers under probation. Good organisations have only lately begun to realise what a difference uniform makes to a body of workers, and now is the time to put this discovery to the proof. We have before pleaded in these columns for the establishment of a service of domestic workers for institutions on the Paris system, which embraces, if we remember rightly, some six grades, each, with its regulation uniform and salary retained, when the workers pass from one institution to another. Without waiting for the establishment of such a public service we would suggest to matrons in dire necessity for want of manual help the trial of part-time workers, wearing uniform when on duty, paid and graded according to efficiency, or length of service. It would not be a difficult or costly experiment. And we believe many married women of a superior class would gladly help to make it a success, if they realised the need.

**☞ It is our invariable practice to pay for all items of news which we may publish. The minimum payment is 5s. Paragraphs of about twenty lines in length—that is to say, of 150 words or less—are preferred. Contributions should be addressed to The Editor, The Hospital, 28 and 29 Southampton Street, Strand, London, W.C. 2, and, to be in time for the current week's issue, should reach him by the first post on Mondays.**

